# Retrospective study on the outcomes and satisfaction with endometrial ablation by bipolar energy (NovaSure^®^) for the treatment of heavy menstrual bleeding

**DOI:** 10.1007/s00404-024-07726-5

**Published:** 2024-09-13

**Authors:** Covadonga Alvarez López, Aida González Paredes, Sonia Martínez Morales, Maria Teresa Aguilar Romero, Mónica Gutiérrez Simón, Jorge Fernández Parra, Alicia Hernández Gutiérrez

**Affiliations:** 1 Department of Gynecology, La Paz University Hospital, IDIPAZ, Pº de la Castellana, 261, 28046 Madrid, Spain; 2 Virgen de Las Nieves Hospital, Av. de Las Fuerzas Armadas, 2, 18014 Granada, Spain; 3 Torrecárdenas Hospital, C. Hermandad de Donantes de Sangre, S/N, 04009 Almería, Spain

**Keywords:** Endometrial ablation, Heavy menstrual bleeding, Bipolar energy, NovaSure^®^

## Abstract

**Purpose:**

To determine the effectiveness, safety, and participant satisfaction with endometrial ablation by bipolar energy (NovaSure^®^) in the treatment of heavy menstrual bleeding (HMB), and to investigate factors associated with poorer outcomes.

**Methods:**

Multicenter retrospective observational study based on medical record review of the outcomes related to endometrial ablation by the bipolar-energy technique procedure to treat HMB in the setting of three university teaching hospitals in Spain.

**Results:**

A total of 333 women were included in the study. Most bipolar-energy ablations were successful (85.12%; *n* = 269 out of 316), with amenorrhea the most frequent outcome (39.6%, *n* = 131 out of 316). The majority of participants had no complications (95.5%; *n* = 317 out of 332), and of those who did, only 2.1% were related to the technique. No further treatment was required for HMB in 82.8% of women (*n* = 274 out of 331), and surgery was avoided in 91.8%; only 5.9% of women underwent ablation-related hysterectomy. In women with previous transverse cesarean sections (CS), 91.0% avoided subsequent surgical treatment. Eighty-six percent of women (*n* = 221 out of 257) were satisfied with the procedure.

**Conclusion:**

Bipolar-energy ablation is very effective and safe for the treatment of HMB and yielded a high rate of participant satisfaction in our setting. The presence of comorbidities or previous CS may slightly reduce the effectiveness of the method, while performing concomitant surgery (mainly curettage) increases the rate of complications. Notably, despite the known increased risk of hysterectomy, most participants with previous CSs who underwent ablation avoided major surgery.

## What does this study add to the clinical work

**Table:** 

Heavy menstrual bleeding interferes with women’s quality of life. NovaSure^®^ is a minimally invasive procedure that reduces bleeding by endometrial ablation effectively and safely with high participant satisfaction.	

## Introduction

Heavy menstrual bleeding (HMB), defined as “excessive menstrual blood loss” that interferes with a woman’s physical, emotional, social, and material quality of life (QoL) [1], is a major reason for medical consultations, accounting for 70% of gynecological visits in peri- and postmenopausal women [2].

When structural abnormalities are not the underlying cause of HMB, the main pharmacologic options are non-steroidal anti-inflammatory drugs (NSAIDs) and antifibrinolytics such as tranexamic acid, with 20–50% efficacy in reducing bleeding, respectively [2]. In Spain, the only hormonal treatments approved in this indication are levonogestrel-releasing intrauterine devices (IUD), and the combined hormonal contraceptive (CHC) pill, which reduce bleeding by 30–94% in volume [2]. However, both are associated with significant drawbacks (side effects, increased cardiovascular risk, etc.) that may contraindicate their use or lead to discontinuation [3].

When pharmacologic treatment fails, or according to participant preference, surgical methods can be applied. The definitive surgical treatment for HMB is hysterectomy. However, it is a major surgery that may involve significant emotional and physical complications, as well as substantial social and economic costs. Thus, in recent years, less invasive surgical procedures have been developed [2].

First-generation techniques consist of extirpation-resection of the endometrium under direct vision using different methods, e.g., endometrial vaporization by rollerball, endometrial resection with monopolar or bipolar resectoscope, or a combination of both. However, these techniques require either previous preparation of the endometrium (administration of GnRh analogs, contraceptives etc.) or it must be performed in the proliferative phase of the cycle. The outcomes are highly dependent on the surgeon’s skills, and the risk of complications is higher than for second-generation techniques [2].

Second-generation techniques destroy the endometrium homogeneously by applying a form of energy directly using an endometrial cavity device. These may be carried out with local anesthesia. Operating room and surgeon training time are much shorter than for first-generation methods.

Among the second-generation ablation techniques, we use bipolar-energy ablation with NovaSure^®^ (Hologic, Inc, Marlborough, MA), which is a system consisting of a single-use three-dimensional, triangular-shaped bipolar ablation device and a radiofrequency controller. This system works until a tissue impedance of 50 ohms is reached, thus destroying the basal layer of the endometrium [4]. This method incorporates a safety test that is performed prior to ablation, so the surgeon cannot continue the procedure if a perforation has occurred. In addition, during the ablation phase and prior to cauterization of the endometrium, a vacuum is generated, which allows good contact to be maintained between the uterine wall and the electrode array, thus avoiding possible energy leaks. A potential disadvantage of bipolar-energy ablation is that, in large uteri, the maximum opening of the electrodes may not cover the entire endometrial surface. In these cases, the generator may detect vacuum defects and stop before the ablation has been successfully completed. Even if the ablation has been completed satisfactorily, a follow-up hysteroscopy may show that not all of the endometrial surface has been cauterized.

The aim of this study was to examine the clinical outcomes, complications and satisfaction associated with the bipolar-energy procedure for endometrial ablation to reduce HMB, as well as to identify possible factors associated with technique failure, with a view to finding solutions to avoid clinical repercussions.

## Methods

### Study design and population

This study was designed as a retrospective observational study on a hospital register of women who underwent bipolar energy endometrial ablation to treat HMB in three university hospitals in Spain: La Paz (Madrid), Torrecárdenas (Almería), and Virgen de las Nieves (Granada). It includes all participants treated since the first procedure was performed in 2009 up to 29 January 2021, the database cut-off date. Women were candidates for the bipolar-energy ablation procedure if: their menstrual flow was so heavy that it affected their QoL (interfered with normal daily activities during their period); hormonal treatment was contraindicated, had failed, or was not desired; and who had completed childbearing. Women who were pregnant, had malignant or premalignant uterine pathology, active pelvic or urinary infection, anatomic conditions that weaken the myometrium (e.g., previous classical cesarean section or transmural myomectomy), or uterine cavity length less than 4 cm were not eligible for the bipolar-energy ablation.

Endometrial ablation performed using Novasure consists of the following steps: (1) Perform cavity measurements (cavimetry), which is the distance from the endometrial fundus to the internal cervical os. (2) Introduce these cavity measures into the device, so that the electrode mesh will adjust to these dimensions, fully covering the endometrial cavity without reaching the endocervical canal; (3) Safety test: an influx of CO2 enters the cavity and, if the gas pressure is maintained for a few seconds, it means that there has been no uterine perforation; thus enabling safe ablation (no energy leaks). (4) Radiofrequency endometrial ablation will begin until the generator detects an impedance of 50 OMH, which will indicate that we have reached the myometrium and automatically stop.

The study was approved by the ethics committees of the three hospitals.

### Collected variables

Outcome variables were collected between 3 and 6 months after the intervention. Possible effectiveness outcomes were categorized as successful (amenorrhea, oligomenorrhea or amenorrhea) or unsuccessful (menorrhagia or chronic pelvic pain syndrome [CPPS]). Satisfaction was initially assessed on a three-point scale as “dissatisfied”, “satisfied” and “completely satisfied”, but for ease of interpretation, it has been recategorized a posteriori as “dissatisfied” and “satisfied”, with the latter including both “satisfied” and “completely satisfied”.

### Calculations

The variables collected are described as absolute and relative frequencies (N, %). ${\chi }^{2}$ tests were used to evaluate the possible association between the dependent variables (i.e., satisfaction, outcomes and complications) and the independent variables, using an alpha significance level of 0.05. Due to the high rate of missing data inherent to the retrospective record-based design, each analysis was carried out on the subpool of participants presenting the data of interest. The reference number of participants is specified in parenthesis for each result.

## Results

Of the 337 participants included in the register, four did not undergo the ablation procedure. In four participants, the procedure was halted due to activation of the safety mechanism of the NovaSure^®^ device: wound dehiscence was detected in one participant with previous cesarean delivery and isthmocele, while no apparent cause was identified in the other three women. However, a uterine depth as measured by hysterometer of > 10 cm (*n* = 2) and intramural myomas (*n* = 1) may have caused the procedure to stall, perhaps due to poor adaptation of the uterine walls to the device.

Thus, only 333 women were included in this analysis. The mean age (standard deviation, SD) of the participants at the time of the procedure was 46.39 (4.36) years. Baseline characteristics are detailed in Table 1.

**Table 1:** Baseline characteristics of the study population

Variable	Number (*N*)	Percentage of evaluated sample (%)
Age		
< 45 years	122	36.6
≥ 45 years	211	63.4
Missing	0	–
Previous vaginal deliveries		
Yes	270	81.6
No	61	18.4
Missing	2	–
Previous abortions		
Yes	71	21.5
No	260	78.5
Missing	2	–
Previous transverse cesarean sections		
Yes	68	20.5
No	264	79.5
Missing	1	–
Comorbid conditions		
None	170	51.4
Hypertension	16	4.8
Hypothyroidism	12	3.6
Obesity	8	2.4
Hypercholesterolemia	3	0.9
Diabetes	2	0.6
Other	72	21.8
More than one comorbid condition	48	14.5
Missing	2	–
Condition involving bleeding risk		
None	289	87.6
Hematologic	28	8.5
Oncologic	3	3.0
Other	10	0.9
Missing	3	–
Ultrasound findings		
Normal	193	59.0
Polyps	65	19.9
Myomas	51	15.6
Hypertrophic endometrium	16	4.9
Malformation	2	0.6
Missing	6	–
Previous treatment for HMB		
None	123	37.7
Progestogen	59	18.1
Myomectomy-polypectomy	37	11.3
NSAID-Tranexamic acid	27	8.3
CHC	2	0.6
Other	3	0.9
More than one treatment	75	23.0
Missing	7	–
Concomitant surgical procedures during NovaSure^®^ intervention		
None	274	82.5
Curettage	36	10.8
Essure^®^	13	3.9
IUD removal	3	0.9
Other	6	1.8
Missing	1	–

*NSAID* Non-steroid antiinflammatory drug,* CHC* Combined hormonal contraceptive,* IUD* Intraunterine device,* HMB* Heavy menstrual bleeding

### Effectiveness

Most bipolar-energy ablations were successful (85.12%; *n* = 269 out of 316), with amenorrhea being the most frequent outcome (39.6%, *n* = 131 out of 316). A small proportion of participants had an unfavorable outcome (14.88%; *n* = 47 out of 316), mainly menorrhagia. Only six participants had CPPS as a result. The outcome was unknown for 17 participants.

**Fig. 1:**
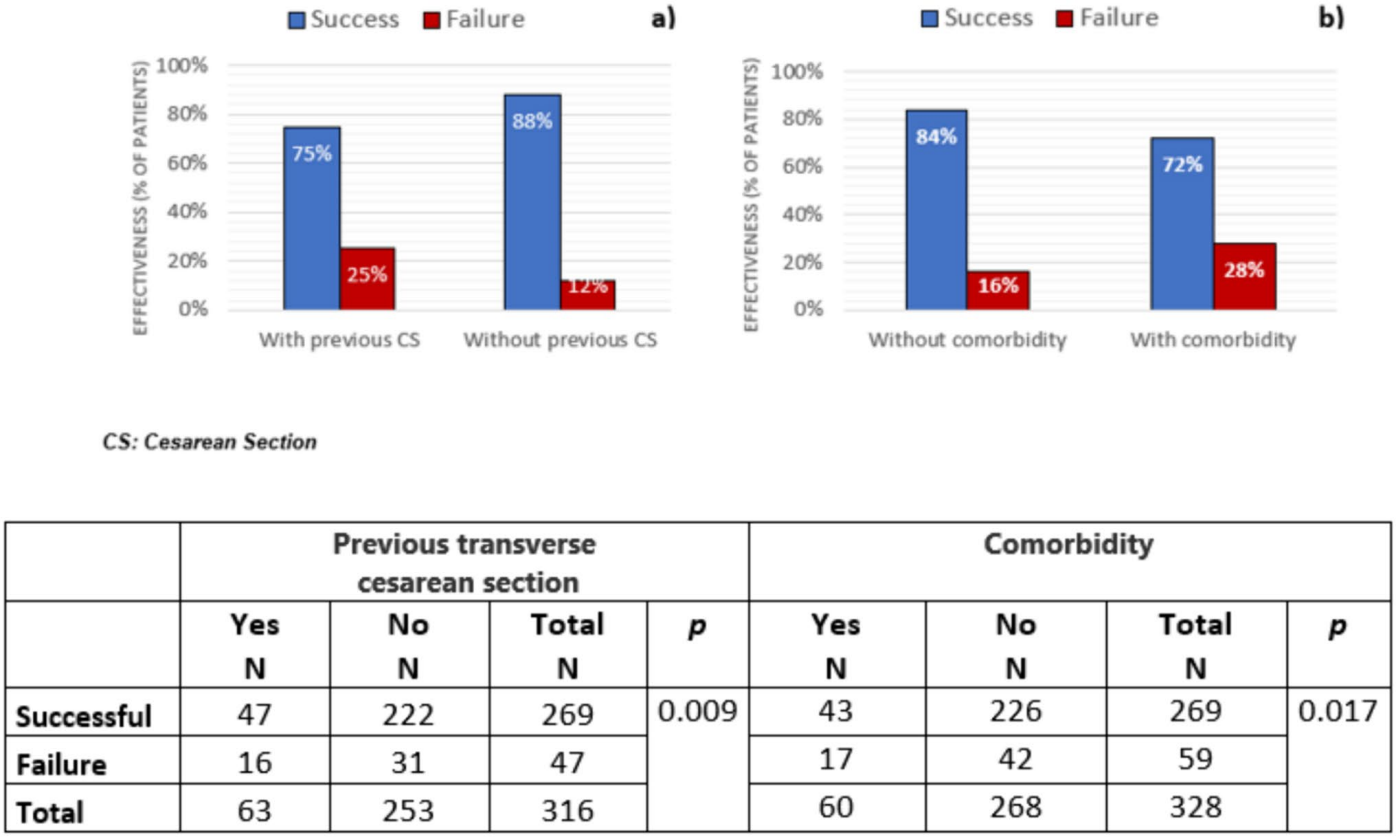
NovaSure^®^ outcomes according to: **a** Previous CS; **b** Comorbid conditions

The majority of participants with previous transverse cesarean sections (CS) had successful ablation outcomes; however, these women experienced a significantly higher frequency of unsuccessful outcomes following the bipolar-energy ablation compared with women who had no previous CS (25% vs. 12%, *p* = 0.009; Fig. 1a). While the proportion of women achieving either amenorrhea or oligomenorrhea was similar in both groups, the rate of women experiencing CPPS among those who had had a previous CS vs. no CS (*n* = 4 vs. *n* = 2; corresponding to 6% vs. 1%) was numerically higher; in addition, more women with previous CS experienced menorrhagia after the procedure than those without previous CS (*n* = 29 vs. *n* = 12; corresponding to 19% vs. 11%).

A significantly higher proportion of women without comorbid conditions had successful procedures (*n* = 226 vs. *n* = 43, corresponding to 84% and 72% of women without and with comorbidities, *p* = 0.017; Fig. 1b). The main comorbidities recorded were hypertension, hyperthyroidism, and obesity; most women with these comorbidities also had successful procedures (Table 2).

**Table 2:** Frequency of ablation outcomes according to the type of comorbidity in participants

	Comorbidity			
	Hypertension	Hypothyroidism	Obesity	Other
Successful	11 (68.8)	9 (75.0)	6 (75.0)	67 (91.8)
Failure	5 (31.3)	3 (25.0)	2 (25.0)	6 (8.2)
Total	16 (100.0)	12 (100.0)	8 (100.0)	73 (100.0)

### Complications

Very few complications occurred during the bipolar-energy ablation, with most participants having no complications at all (95.2%; *n* = 317 out of 333). Among women who did experience complications, five had incomplete ablation (1.5%), five developed an infection (1.5%), four had a false passage created (1.2%), one experienced perforation (0.3%) and one had hemorrhage (0.3%).

It should also be noted that, when we studied the participants in whom the endometrial ablation was not complete, we observed good results in all cases, with a high degree of satisfaction (See Table 3).

**Table 3:** Characteristics and outcomes of participants with incomplete ablation

Participant	Cavimetry	Ablation time	Portion of ablated endometrium according to post-interventional hysteroscopy	Outcome	Satisfaction
1	7.5 cm	30 s	Upper 2/3	Amenorrhea	Very satisfied
2	6 cm	55 s	Entire cavity	Oligomenorrhea	Very satisfied
3	9.5 cm	45 s	Upper ½	Decrease in bleeding	Satisfied
4	5 cm	45 s	Upper 2/3	Eumenorrhea	Very satisfied
5	8.5 cm	–-	Front and back side	Oligomenorrhea	Satisfied

Hypothesis testing showed that significantly more participants with previous CS experienced false passage creation with respect to women without previous CS (*n* = 3 vs. *n* = 1; corresponding to 0.044% vs. 0.004%; *p* = 0.017).

The low number of complications recorded occurred more frequently in participants undergoing a concomitant-surgical procedure (7.4% of participants undergoing a concomitant procedure [4 out of 54] vs. 4.6% of those not undergoing any other procedure [12 out of 262]); however, this difference was not statistically significant. The number of participants experiencing complications in relation to the surgical procedure carried out is detailed in Table 4.

**Table 4:** Count of ablation complications according to concomitant surgery type

Complication	Concomitant surgery type			
	Curettage	Essure^®^ placement	IUD removal	Other
False line	1	0	0	0
Infection	1	0	0	0
Hemorrage	1	0	0	0
Perforation	0	1	0	0
None	33	12	3	6

The concomitant-surgical intervention most frequently associated with post-surgical complications in our population was curettage, and included one infection, one false passage creation, and one hemorrhage.

### Need for postoperative treatment

Most participants required no further treatment for HMB after ablation (84.0%, *n* = 257 out of 306). More invasive surgery was subsequently required in 5.9% of women (*n* = 18 out of 306); one participant underwent a re-ablation procedure (0.3%); and 14.4% still needed pharmacologic treatment post procedure (*n* = 44 out of 306). This variable was assessed at the database closure (2021).

### Satisfaction

Most participants stated they were satisfied or fully satisfied with the bipolar-energy ablation (86.0%; *n* = 221 out of 257).

**Fig. 2:**
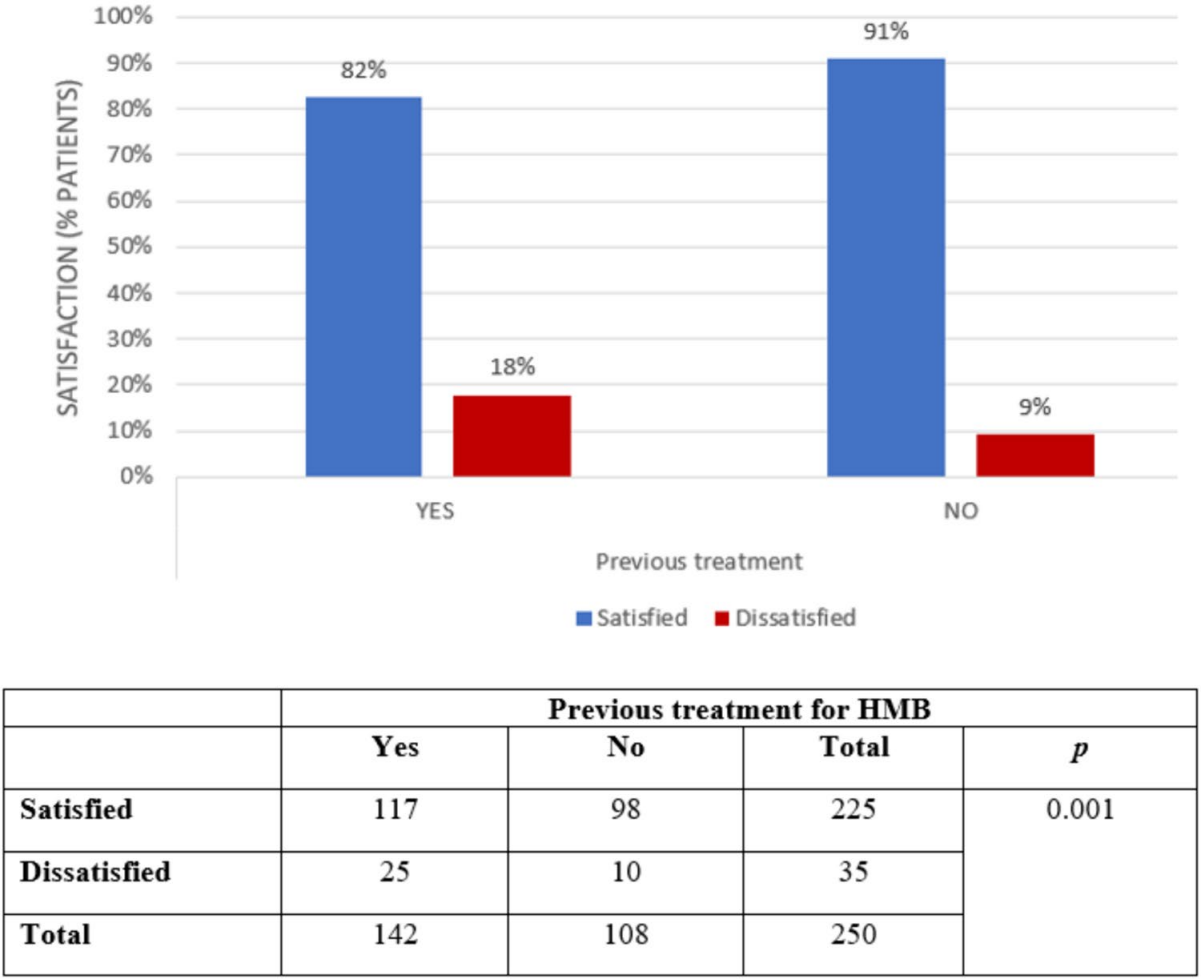
Satisfaction of participants according to previous HMB treatments

Having received no previous treatments for HMB was significantly associated with a higher proportion of women satisfied with the bipolar-energy ablation with respect to women having received previous treatments (*p* = 0.001; Fig. 2).

## Discussion

This is the first study to analyze a large number of participants in Southern Europe who had undergone the bipolar-energy endometrial ablation to reduce HMB. Our analysis of the data from 333 women found that this method was very effective in reducing bleeding, had a good safety profile with very few complications recorded, a very rare need for post-procedure treatments or interventions, and generally high satisfaction. Surgery was avoided in a significant number of participants with previous CSs, which is a great advantage for these women, who have a high surgical risk.

### Effectiveness

Our real-world evidence is consistent with previous studies conducted in other countries. Various single-center studies with smaller samples than ours (*n* = 50–125) carried out in the UK and the US showed a success rate of 90%-97% for the bipolar-energy ablation [5–7], and a similar rate (95.2%) was recorded for a large single-center study in China (*n* = 2152) [8]. Although our effectiveness rate seems slightly lower, it should be taken into consideration that, in Spain, ablation is often applied only after several other methods have failed. Thus, our study population may have more severe or resistant forms of HMB. Indeed, having received previous treatment for HMB was significantly associated with a lower degree of participant satisfaction in our study.

Another circumstance to bear in mind is that we regarded the onset of CPPS as “procedure failure”. This may be considered a confounder, in that CPPS is a relatively common complication of CS. According to a meta-analysis published by Weibel et al. [9]*,* 15% and 11% of the women who had undergone previous CS developed CPPS at 3 and ≥ 12 months, respectively. In our study, 5.8% of participants with previous CS reported CPPS, which is lower than the general population of women with previous CS who have not undergone endometrial ablation. In fact, previous CS was significantly associated with lower success rates. Accordingly, considering CPPS as a “procedure failure” may have contributed to the apparently lower success rates observed in participants with previous CS. To corroborate this, other studies that did not find an association between previous CS and lower success rates [10, 11] only considered menorrhagia in their definition of ablation failure.

### Complications

In line with previous studies published in different countries, the rate of complications associated with bipolar-energy ablation was very low in our study [5–8].

Although we have reported an overall complication rate of 4.5%, the actual rate of adverse events associated with the ablation technique was 2.1% (*n* = 5 infections, *n* = 1 perforation, *n* = 1 hemorrhage). In addition, 1.2% of participants experienced false passage creation; however, this should not be considered a complication associated with ablation per se*,* but with the previous cervical dilatation. We believe this percentage may be reduced by performing a previous diagnostic hysteroscopy, allowing for direct vision in those women who may be at risk of complicated dilatation, such as those with no previous vaginal deliveries.

We also reported that 1.5% of women had incomplete ablation. However, what we call incomplete ablation refers to a problematic procedure, in which, when the probe was correctly placed and adjusted to the uterine fundus, the destruction of a good portion of the endometrium was obtained. Our participants achieved significant symptom improvement and a high degree of satisfaction with the procedure, despite being unable to ablate the entire endometrium.

Finally, as we gain experience in the use of this technique, we have realized that many of the cases diagnosed as endometritis were really a common physiologic inflammatory reaction to the procedure. Some women present a yellowish discharge also described by Xie et al*.* [8] and low-grade fever in the first few days after ablation not associated with any serological alteration. This process is self-limiting and does not require any specific treatment. Therefore, we believe that the 1.5% of infections that we recorded may actually be lower.

In our study, factors significantly associated with more complications were previous CS and performing the ablation concomitant with other surgical procedures. In the literature, CS have not been related to an increase in complications. In Kahn et al. [10], the only intraoperative complication was uterine perforation: two cases occurred in women in the CS group (1.2%) and two in women in the without-CS group (0.4%). All perforations that occurred were fundal in location and remote from the low-segment uterine scar. One study reported outcomes in women who concomitantly underwent NovaSure^®^ with the Essure^®^ procedure, in which the authors reported 100% effectiveness of ablation and satisfactory placement of the sterilization device (95%) without complications [12]. In our study, one woman experienced perforation during ablation and insertion of Essure^®^.

The concomitant-surgical procedure most frequently associated with complications was curettage. Thus, to reduce ablation-complication rates, we suggest that this procedure be avoided in the same surgical session. We also recommend performing a previous hysteroscopy which would: (1) allow cervical dilatation to be performed under direct vision, avoiding the risk of false passage creation; (2) provide complete endometrial visualization, facilitating diagnosis of cavity conditions that may make ablation difficult or impossible; and (3) permit endometrial biopsy to be carried out, if needed.

### Need for subsequent treatment

With the consistently high effectiveness of the procedure, only a few participants needed further treatment for HMB. In particular, ablation-related hysterectomy was carried out in 5.9% of participants and only one woman underwent a re-ablation procedure (0.3%), with our re-intervention rate being 6.1%, lower than the 9.4% presented by Ghoubara et al*.* [13] On the other hand, previously reported data were lower for the hysterectomy rate: Kalkat et al. [6] reported that 2.0% of participants underwent hysterectomy, Scordalakes et al. [7] noted 4.1%, and Xie et al. [8], 1.9%. When interpreting our hysterectomy data, it should also be taken into account that many women were followed for more than 5 years. Hysterectomy data collection for all included participants was performed 1 year after the database cut-off date. Oderkerk et al. [14] found that the risk of hysterectomy after endometrial ablation seems to increase from 4.3% after 1 year to 12.4% after 5 years.

As previously mentioned, in Spain, ablation is often the second, third or even subsequent line of treatment for women with HMB. Many women undergoing ablation in our setting are those in whom hormonal IUD has failed, perhaps due to a large cavity. Indeed, approximately half of the participants who eventually underwent hysterectomy had several previous treatments (*n* = 12).

Thus, most women treated with endometrial ablation had exhausted medical treatment options for HMB. Not only did we avoid surgery in 84.0% of the participants, who required no further treatment, but it also proved unnecessary in 14.4% of the women who, while not achieving definitive success, could subsequently be controlled with medical treatment only. Accordingly, 98.4% of our participants achieved control of their HMB without major surgery. In addition, 91% of women with previous CS, who are at higher risk for reinterventions, avoided surgery.

### Satisfaction

Most women in this study were satisfied or very satisfied with the NovaSure^®^ procedure (86.0%; *n* = 221 out of 257). Other studies assessing participant satisfaction have reported similar rates: Elmardi et al. reported 87.0% [5], Kalkat et al., 86.0% [6], Ghoubara et al.[13] 81.7%, and Scordalakes et al., 94.0% [7]. A patient-reported outcome and satisfaction survey in 115 women in Scotland reported a degree of success of 94.4% and 90.4% satisfaction with the bipolar-energy endometrial ablation^®^ (15).

In our subanalysis, an independent factor associated with a higher degree of satisfaction was having had no previous treatment for HMB.

### Limitations

The main limitations of this study are inherent to its retrospective design. Not all variables had been filled out for all participants, and thus subanalyses relied only on women who had the necessary variables recorded, with subsequent loss of information. Even so, this remains the first study of its kind to be carried out in Spain and with a good sample size.

## Conclusions

Bipolar-energy endometrial ablation is very effective and safe for the treatment of HMB and yields a high rate of satisfaction in our setting, especially avoiding surgery in high-risk participants (women with previous CS).

The presence of comorbidities or history of CS may slightly reduce the effectiveness of the method, while performing concomitant surgery (mainly curettage) increases the rate of complications.

Diagnostic hysteroscopy prior to bipolar-energy endometrial ablation may prevent complications and improve outcomes.

It would be interesting to investigate the impact of moving ablation to an earlier treatment line on the bipolar-energy procedure outcomes.

## Data Availability

The original study data are available from the corresponding author upon request. All performed analyses are available in the manuscript.
